# Investigation of *Streptococcus salivarius*-mediated inhibition of pneumococcal adherence to pharyngeal epithelial cells

**DOI:** 10.1186/s12866-016-0843-z

**Published:** 2016-09-29

**Authors:** Jayne Manning, Eileen M. Dunne, Philip A. Wescombe, John D. F. Hale, E. Kim Mulholland, John R. Tagg, Roy M. Robins-Browne, Catherine Satzke

**Affiliations:** 1 Pneumococcal Research, Murdoch Childrens Research Institute, The Royal Children’s Hospital, Parkville, VIC Australia; 2 Infectious Diseases and Microbiology, Murdoch Childrens Research Institute, The Royal Children’s Hospital, Parkville, VIC Australia; 3 Department of Microbiology and Immunology at the Peter Doherty Institute for Infection and Immunity, The University of Melbourne, Parkville, VIC Australia; 4 Blis Technologies Ltd, Dunedin, New Zealand; 5 London School of Hygiene and Tropical Medicine, London, UK; 6 Department of Microbiology and Immunology, University of Otago, Dunedin, New Zealand; 7 Department of Paediatrics, The University of Melbourne, Parkville, VIC Australia

**Keywords:** Probiotics, Probiotic mechanisms, Respiratory tract, *Streptococcus salivarius*, *Streptococcus pneumoniae*, Pneumococcus, Colonisation, Adherence

## Abstract

**Background:**

Pneumococcal adherence to the nasopharyngeal epithelium is a critical step in colonisation and disease. The probiotic bacterium, *Streptococcus salivarius*, can inhibit pneumococcal adherence to epithelial cells in vitro. We investigated the mechanism(s) of inhibition using a human pharyngeal epithelial cell line (Detroit 562) following pre-administration of two different strains of *S. salivarius*.

**Results:**

Whilst the bacteriocin-encoding megaplasmids of *S. salivarius* strains K12 and M18 were essential to prevent pneumococcal growth on solid media, they were not required to inhibit pneumococcal adherence. Experiments testing *S. salivarius* K12 and two pneumococcal isolates (serotypes 19F and 6A) showed that inhibition of 19F may involve *S. salivarius*-mediated blocking of pneumococcal binding sites: a negative correlation was observed between adherence of K12 and 19F, and no inhibition occurred when K12 was prevented from contacting epithelial cells. K12-mediated inhibition of adherence by 6A may involve additional mechanisms, since no correlation was observed between adherence of K12 and 6A, and K12 could inhibit 6A adherence in the absence of cell contact.

**Conclusions:**

These results suggest that *S. salivarius* employs several mechanisms, including blocking pneumococcal binding sites, to reduce pneumococcal adherence to pharyngeal epithelial cells. These findings extend our understanding of how probiotics may inhibit pneumococcal adherence and could assist with the development of novel strategies to prevent pneumococcal colonisation in the future.

**Electronic supplementary material:**

The online version of this article (doi:10.1186/s12866-016-0843-z) contains supplementary material, which is available to authorized users.

## Background

*Streptococcus pneumoniae* (the pneumococcus) commonly colonises the nasopharynx of healthy humans, especially young children. Carriage is considered a prerequisite for pneumococcal disease and facilitates the transmission of pneumococci throughout communities [1]. Dissemination of pneumococci from the nasopharynx to other body sites can give rise to diseases such as meningitis, sepsis, pneumonia, and otitis media. An estimated 800,000 children under the age of five die from pneumococcal infections each year, with most deaths occurring in low-income countries where carriage rates are especially high [2]. Strategies targeting the reduction of pneumococcal colonisation could potentially reduce this burden of disease.

Current pneumococcal conjugate vaccines (PCVs) induce protection against 10–13 of the most common disease-causing serotypes via the induction of anti-capsular antibodies. Although PCVs have successfully reduced carriage and disease caused by vaccine serotypes, they are expensive to produce and have led to an increase in colonisation by non-vaccine serotypes (serotype replacement) [3]. In recent years, there has been increased interest in the use of probiotics, which are defined as live microorganisms that can confer a health benefit to the host, to reduce pathogen colonisation and respiratory tract infections [4]. Proposed mechanisms of probiotic action include inhibition of pathogen colonisation via competition for binding, direct inhibition due to the activity of secreted antimicrobial molecules and the induction of immunomodulatory effects in the host [5–9].

*Streptococcus salivarius* is a member of the respiratory tract microbiota and has been commercially available as an oral probiotic for more than a decade [10]. Small clinical trials have shown that administration of *S. salivarius* strains K12 and M18 can reduce the occurrence of tonsillitis and otitis media [11] and reduce dental plaque levels in children [12], as well as treat halitosis in adults [13]. Several in vitro studies have found that *S. salivarius* can prevent the growth of a range of respiratory pathogens, including the pneumococcus, through production of megaplasmid-encoded bacteriocins and bacteriocin-like inhibitory substances (BLIS) [7, 14–16]. However, the mechanisms by which they inhibit pathogen adherence in vivo are unknown. We have previously shown that *S. salivarius* K12 can inhibit pneumococcal adherence to a human epithelial cell line (CCL-23) [17]. Here, we demonstrate that the same phenomenon is observed in Detroit 562 pharyngeal epithelial cells and investigate the inhibitory mechanisms involved, including the role of the *S. salivarius* megaplasmid. Our results suggest that *S. salivarius* K12 inhibits pneumococcal adherence by blocking pneumococcal binding sites, although other mechanisms such as direct interference through the action of secreted molecules may also contribute.

## Methods

### Bacterial strains, cell lines and culture conditions

Bacterial isolates are described in Tables 1 and 2. Pneumococcal isolates were selected to represent a range of serotypes and based on their ability to adhere to human epithelial cells. *S. salivarius* isolates were sourced from Blis Technologies Ltd, New Zealand. All isolates were cultured at 37 °C in 5 % CO_2_ on horse blood agar (HBA; Thermo Fisher Scientific) plates, in Todd-Hewitt broth (THB; Oxoid), or THB supplemented with 0.5 % (w/v) yeast extract (THY; Becton Dickinson). Deferred antagonism testing was carried out on BaCa (Columbia agar base; Life Technologies Ltd.) plates supplemented with human blood (5 %, v/v) and CaCO_3_ (0.1 %, w/v) except where noted.

**Table 1:** *Streptococcus salivarius* isolates used in this study

*S. salivarius* isolate	Known bacteriocins
K12	SalA, SalB
K12 ^mp−^	–
M18	SalA, Sal9, SalM
M18 ^mp−^	–
A234	SalA
NR	SalB
T18A	SalA, SalB
T30A	Unknown
Min5	SalA, SalB
20P3	SalA

SalA: salivaricin A [36]; SalB: salivaricin B [23]; Sal9: salivaricin 9 [37]; SalM: salivaricin M [24]; mp: megaplasmid

**Table 2:** Pneumococcal isolates used in this study

Pneumococcal isolate	Serotype	Origin
PMP1081	1	Australia
PMP278	3	Fiji
PMP241	4	South Africa
PMP812	5	Bangladesh
PMP6 (ATCC® 6305™)	5	Germany
PMP1043	6A	USA
PMP17	6A	Fiji
PMP434	6B	Fiji
PMP437	6C	Fiji
PMP1086	7F	Australia
PMP296	9V	Fiji
PMP130	14	Fiji
PMP222	18C	South Africa
PMP843	19F	USA
PMP292	19A	Fiji
PMP283	22F	Unknown

The Detroit 562 pharyngeal epithelial carcinoma cell line (ATCC CCL-138) was maintained in RPMI 1640 (Sigma-Aldrich) supplemented with 10 % (v/v) foetal bovine serum (FBS, Thermo Fisher Scientific). Monolayers were released by incubation with 0.25 % trypsin/EDTA (0.25 % (w/v) trypsin, 0.1 mM EDTA, Life Technologies) for 10 min at 37 °C in 5 % CO_2_ and seeded into 24-well trays at a concentration of 1.5 × 10^5^ cells/well in RPMI supplemented with 5 % FBS for use in adherence assays.

### Deferred antagonism testing

Testing for deferred antagonism was performed as described previously [18]. Briefly, overnight THB cultures of *S. salivarius* (producer strain) were used to inoculate a 1-cm-wide line down the middle of a human blood agar plate (as described above). Plates were incubated for 18 h at 37 °C in 5 % CO_2_, *S. salivarius* growth was removed, and the plate inverted over a chloroform-soaked cloth for 30 min to kill any remaining bacteria. Residual chloroform was then evaporated by exposure of the agar surface to air for 30 min. Overnight THB cultures of pneumococcal indicator strains were then streaked at right angles to the previously grown *S. salivarius* and incubated for a further 12–16 h. Plates were examined for growth where *S. salivarius* had previously grown, and prevention of pneumococcal growth recorded as + (observed) or – (not observed).

### Adherence assays

Adherence assays were performed as described previously [17] with several modifications. D562 cells seeded into 24-well trays were washed twice with Hanks buffered salt solution (HBSS; Gibco®, Life Technologies) before the addition of 500 μl/well RPMI without serum. Bacteria were grown to log phase in THY (*S. salivarius*: 1.5 h, pneumococci: 3 h) and resuspended in 0.85 % (w/v) NaCl (Merck) to ~1.5 × 10^9^ CFU/ml. A 10-fold dilution series of *S. salivarius* was prepared in 0.85 % NaCl and 10 μl aliquots of high (~1.5 × 10^7^ CFU/well), medium (~1.5 × 10^6^ CFU/well), and low (~1.5 × 10^5^ CFU/well) concentrations of bacteria were administered to duplicate wells before centrifugation at 114 × *g* for 3 min to promote bacterial adherence to the cell monolayer. Plates were incubated at 37 °C in 5 % CO_2_ for 1 h. Pneumococci were prepared as above and added to all wells at a concentration of ~1.5 × 10^6^ CFU/well (MOI of 10 pneumococci: 1 D562 cell) 1 h after *S. salivarius*. Centrifugation was performed as above and plates were incubated for a further 1 h. Cells (and adherent bacteria) were then washed three times with HBSS and harvested after the addition of 200 μl 0.25 % trypsin/EDTA to each well, incubation for 10 min at 37 °C in 5 % CO_2,_ and addition of 800 μl of THY to each well with mechanical disruption. Lysates were stored at −20 °C until tested. Pneumococcal adherence (genome copies/well) was determined by quantitative real-time PCR (qPCR) targeting *lytA* (see below). The effect of bacterial infection on D562 cells was monitored microscopically and no significant cytopathic effects were observed during the adherence assay conditions. The effect of *S. salivarius* on pneumococcal adherence was calculated by normalising all treatments against wells containing pneumococci alone. The ability of the pneumococcal isolates to invade cells was measured by incubating cells with media containing 10 μg/ml penicillin and 200 μg/ml gentamicin for 15 min after incubation with pneumococci for 1 h (Additional file 1), confirming the validity of this model to investigate adherence. The adherence of *S. salivarius* to D562 cells at the time of pneumococcal administration was calculated following incubation of ~1.5 × 10^6^ CFU/well bacteria for 1 h at 37 °C in 5 % CO_2_. Cells were washed three times with HBSS to remove non-adherent bacteria and harvested as above. CFU/well was determined by viable count on HBA plates and expressed as mean % adherence ± standard deviation compared to the inoculum added.

We performed preliminary experiments to determine whether heparin, which can inhibit pneumococcal adherence to epithelial cells in vitro by competitively binding to glycosylaminoglycans [19], could also inhibit *S. salivarius* adherence to D562 cells. Cells were incubated with 100 U/well of heparin (Pfizer) for 1 h before the addition of *S. salivarius* as above and results expressed as % adherence compared to *S. salivarius* without heparin. *S. salivarius* adherence to D562 cells over time was measured by qPCR targeting *dex* (see below).

We also investigated whether non-adherent *S. salivarius* had an effect on pneumococcal adherence. To do this, adherence assays were performed as described above, except that wells were washed three times with HBSS to remove unbound *S. salivarius* and fresh medium was added after *S. salivarius* pre-administration and before the addition of pneumococci. To determine the inhibitory effect of *S. salivarius* in the absence of cell contact, *S. salivarius* was pre-administered to D562 cells via permeable transwell inserts with a pore size of 0.4 μm (Corning, In vitro Technologies). To further investigate the mechanisms involved in the inhibition of pneumococcal adherence, preparations of K12 were: a) heat-killed by incubating at 70 °C for 30 min with their lack of viability confirmed by the absence of growth on HBA; b) enzymatically treated to remove outer surface proteins and carbohydrates of *S. salivarius* K12 by (i) resuspending K12 inocula in a final concentration of 5 mg/ml pronase E [20], or (ii) by the addition of 5 μl of 1 mg/ml sodium periodate [21] to K12 inocula prepared in 0.85 % NaCl for 15 min at 37 °C in 5 % CO_2_, and c) reducing K12 protein synthesis by resuspending K12 inocula in 2 mg/ml spectinomycin and incubating for 30 min at 37 °C in 5 % CO_2_. Following all treatments, K12 inocula were washed twice in 0.85 % NaCl prior to use in adherence assays.

### qPCR

DNA extraction and pneumococcal qPCR assays were performed as described previously [17]. Primers (Sigma Aldrich) and dual-labelled probes (Eurogentec) were designed to detect chromosomal (dextranase gene, *dex*) and megaplasmid (mp) target sequences in *S. salivarius*. Primer/probe sequences and final concentrations were as follows: dexF2: TGAAGCAGATAACTTGGTGGTG (300 nM); dexR2: CTCTCTGCTGGCACAGCTT (300 nM); dex probe2: HEX-AGAAGTAGGTCCATCATCTGCC-3′-BHQ-1 (75 nM); mpF: AAGCCTTGTGCATCGACTCT (200nM); mpR: AACCAAGACGCGACTGTTGA (200 nM); mp probe: FAM-TGACCCTTTTTGTTGGTCGT-3′-BHQ-1 (300 nM). Specificity of both assays was confirmed by sequencing the amplified product and testing against a panel of closely related streptococcal species (Additional file 2: Table S1). qPCR reactions were carried out in duplicate using Agilent Brilliant III master mix (Agilent Technologies, Integrated Sciences), containing 1 μl of template DNA in 25 μl final volume using a Stratagene Mx3005P qPCR machine (Agilent Technologies). The cycling conditions were: 1 × 3 min at 95 °C, 40 × 20 s at 95 °C followed by 20 s at 60 °C. Pneumococcal and *S. salivarius* DNA was quantified using standard curves of reference strain isolates (ATCC 6305 and K12, respectively) and bacterial loads were calculated as described previously [22], based on 1 pg of genomic DNA being equivalent to 447.4 pneumococcal cells and 422.1 *S. salivarius* cells (assuming one genome per cell, and one copy of the target gene per genome).

### Statistical analysis

Data were analysed using Prism 6.0d (GraphPad Software, Inc.) and Excel (Microsoft). Student’s t-test was used to compare pneumococcal adherence in all treatment groups. Spearman’s rank test was used to correlate pneumococcal and *S. salivarius* K12 adherence in adherence assays. A *p* value of < 0.05 was considered statistically significant for all assays. For all experiments *n* ≥ 3 unless otherwise stated.

## Results

### *S. salivarius* reduces pneumococcal adherence to pharyngeal epithelial cells

Previous studies in our laboratory demonstrated that *S. salivarius* K12 inhibits pneumococcal adherence to CCL-23 human epithelial cells in vitro, with the strongest effect seen when K12 was added before pneumococci (pre-administration). In this study, we investigated the mechanisms underlying this inhibition using a cell line more relevant to the pneumococcal ecological niche of the nasopharynx, Detroit 562 human pharyngeal epithelial cells (D562), and two isolates representing serotypes that commonly colonise (6A [PMP1043] and 19F [PMP843]). Pneumococcal adherence was measured following pre-administration of commercial probiotic *S. salivarius* strains K12 or M18 at high, medium and low doses.

**Fig. 1:**
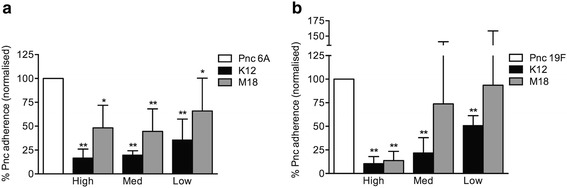
Pneumococcal adherence of serotypes 6A (**a**) and 19F (**b**) to pharyngeal epithelial cells following pre-administration of *S. salivarius.* Approximately 1.4 × 10^6^ CFU pneumococci were added to D562 monolayers at an MOI of 11:1. Pneumococcal adherence was determined when incubated with pneumococci alone (Pnc, normalised to 100 %), or pre-incubated for 1 h with *S. salivarius* K12 or M18 at high (~1.6 × 10^7^ CFU), medium (~1.6 × 10^6^ CFU), or low (~1.6 × 10^5^ CFU) doses. Data are mean + SD; *n* ≥ 3. * indicates *p* < 0.05, ** indicates *p* < 0.001 when compared to Pnc alone (Student’s *t*-test)

All doses of K12 significantly inhibited 6A and 19F adherence (*p* < 0.001, Fig. 1). All doses of M18 inhibited 6A adherence (high: *p* < 0.001, medium: *p* < 0.001, low: *p* = 0.023, Fig. 1a), but only the high dose inhibited 19F adherence (*p* < 0.001, Fig. 1b). K12 adherence to D562 cells was higher than that of M18 (532.3 ± 86.49 % vs. 12.5 ± 8.14 %, *p* < 0.001). Heparin, which is known to inhibit pneumococcal adherence to epithelial cells by competing for binding to cell surface glycosylaminoglycans [19], did not affect *S. salivarius* K12 adherence to D562 cells (115.8 ± 27.81 %, *p* = 0.380, Student's *t*-test).

**Fig. 2:**
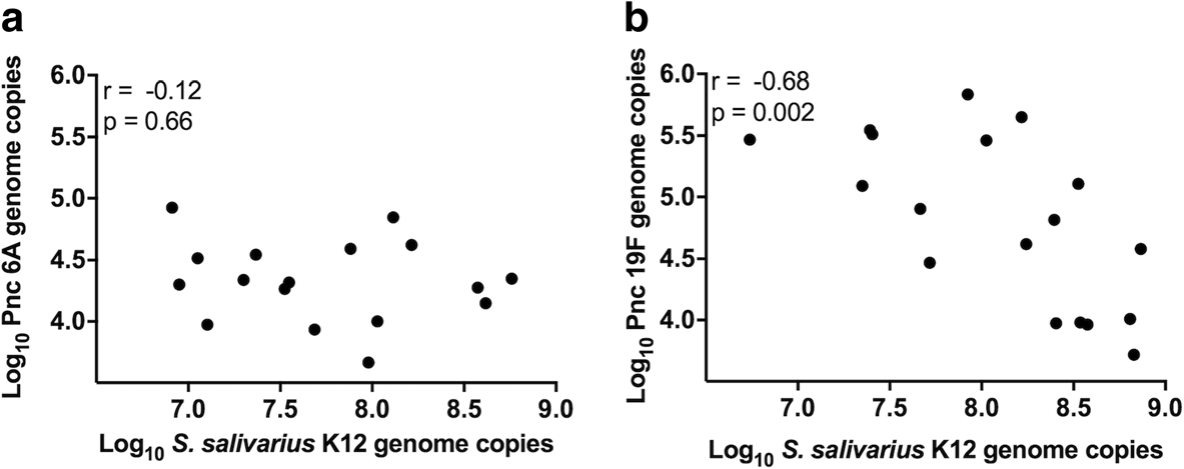
Correlation of adherence of *S. salivarius* K12 and pneumococcal serotype 6A (**a**) and 19F (**b**) adherence to pharyngeal epithelial cells. Adherence of *S. salivarius* and pneumococci to D562 monolayers was determined following pre-incubation for 1 h with high (~1.6 × 10^7^ CFU), medium (~1.6 × 10^6^ CFU), or low (~1.6 × 10^5^ CFU) doses of *S. salivarius* K12 and subsequent incubation with ~1.4 × 10^6^ CFU pneumococci for 1 h. *r* = Spearman rank correlation coefficient

K12 adherence showed significant negative correlation with 19F adherence but not with 6A adherence (19F: *r* = −0.68, *p* = 0.002; 6A: *r* = −0.12, *p* = 0.66; Fig. 2). Significant inhibition of pneumococcal adherence was also observed following removal of non-adherent K12 by washing prior to the addition of pneumococci (Direct + wash, Table 3: all doses *p* < 0.001).

**Table 3:** Pneumococcal adherence to pharyngeal epithelial cells following pre-administration of *S. salivarius* K12 by various methods

		Pneumococcal adherence^a^					
		Direct		Transwell		Direct + wash	
Pneumococcal serotype	K12 Dose^c^	% (95 % CI)	*p* value^b^	% (95 % CI)	*p* value^b^	% (95 % CI)	*p* value^b^
6A	High	16.6 (6.8, 26.5)	<0.001	90.5 (23.9, 157.1)	0.698	25 (12.5, 37.4)	<0.001
	Medium	19.6 (14.7, 24.4)	<0.001	61.5 (18.4, 104.5)	0.049	45.2 (21.9, 68.5)	<0.001
	Low	35.4 (12.3, 58.5)	<0.001	44.1 (22.8, 65.5)	<0.001	45.1 (29.1, 61.1)	<0.001
19F	High	10.4 (2.5, 18.4)	<0.001	84.7 (33.6, 135.7)	0.338	41.8 (17.6, 66.4)	<0.001
	Medium	21.8 (1.7, 41.8)	<0.001	121.3 (16.5, 226.2)	0.581	31.4 (13.2, 49.6)	<0.001
	Low	50.7 (33.8, 67.5)	<0.001	102.2 (33.7, 170.5)	0.933	45.3 (16.7, 74.0)	<0.001

^a^Direct: pre-administration of K12 directly onto D562 monolayer; Transwells: pre-administration of K12 via transwell inserts; Direct + wash: pre-administration of K12 directly onto D562 monolayers followed by a washing step to remove non-adherent K12

^b^ *p* values calculated from pneumococcal adherence alone compared to pneumococcal adherence following different methods of K12 pre-administration (Student’s t test, *p < 0.05)

^c^High: ~1.5 × 10^7^ CFU/ml; Medium: ~1.5 × 10^6^ CFU/ml and Low: ~1.5 × 10^5^ CFU/ml

### The K12 megaplasmid is essential to prevent pneumococcal growth but not adherence

The megaplasmids of *S. salivarius* K12 and M18 encode bacteriocins that prevent the growth of a range of bacterial species on solid media [14, 16, 23, 24], but their roles in inhibition of pneumococcal growth or adherence are unknown. We compared the ability of K12, M18, their megaplasmid-negative derivatives, and a panel of *S. salivarius* strains to inhibit the growth of 15 pneumococcal isolates by deferred antagonism. *S. salivarius* strains K12, M18, A234, NR, T18A and Min5 inhibited all of the pneumococcal isolates tested (Table 4). Neither megaplasmid-negative strain (K12^mp−^ or M18^mp−^) or strain T30A prevented the growth of any of the pneumococcal isolates tested (Table 4).

**Table 4:** Deferred antagonism of *S. salivarius* strains against a panel of pneumococcal isolates

		*S. salivarius* producer strain									
Pneumococcal test strain	Serotype	K12	K12 ^mp−^	M18	M18 ^mp−^	A234	NR	T18A	T30A	Min5	20P3
PMP1081	1	+	−	+	−	+	+	+	−	+	+
PMP278	3	+	−	+	−	+	+	+	−	+	+
PMP241	4	+	−	+	−	+	+	+	−	+	−
PMP812	5	+	−	+	−	+	+	+	−	+	+
PMP1043	6A	+	−	+	−	+	+	+	−	+	−
PMP17	6A	+	−	+	−	+	+	+	−	+	+
PMP434	6B	+	−	+	−	+	+	+	−	+	+
PMP437	6C	+	−	+	−	+	+	+	−	+	+
PMP1086	7F	+	−	+	−	+	+	+	−	+	+
PMP296	9V	+	−	+	−	+	+	+	−	+	+
PMP130	14	+	−	+	−	+	+	+	−	+	−
PMP222	18C	+	−	+	−	+	+	+	−	+	+
PMP292	19A	+	−	+	−	+	+	+	−	+	−
PMP843^a^	19F	+	−	+	–	NT	NT	NT	NT	NT	NT
PMP283	22F	+	−	+	−	+	+	+	−	+	+

“+” indicates inhibition of pneumococcal growth and “−" indicates no inhibition, *n* = 2

*NT* not tested

^a^performed on horse blood agar plates

**Fig. 3:**
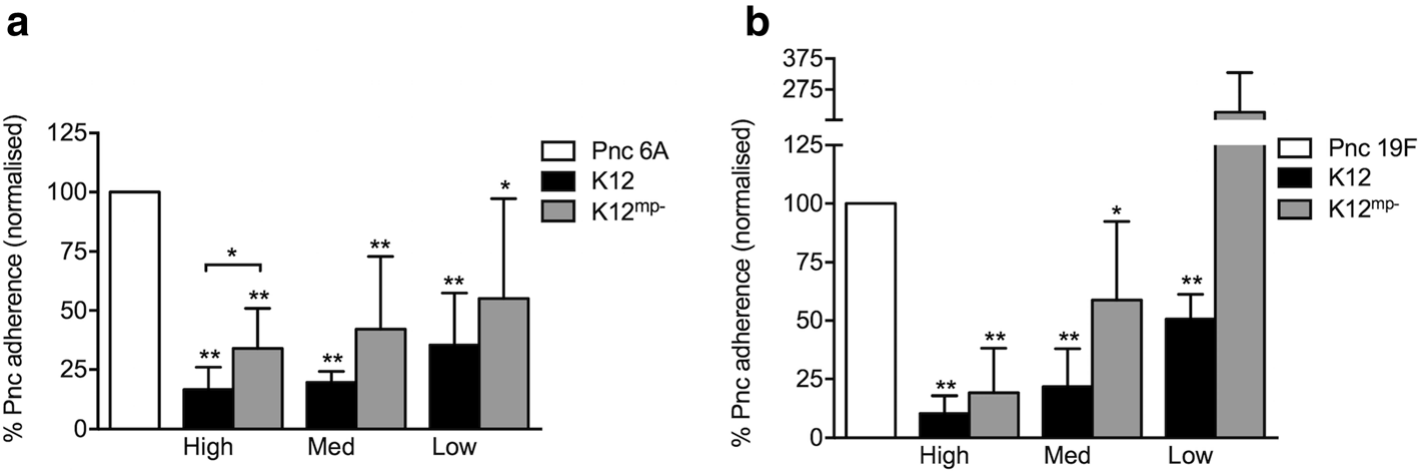
Adherence of pneumococcal serotypes 6A (**a**) and 19F (**b**) to pharyngeal epithelial cells following pre-administration of megaplasmid positive (K12) and negative (K12 ^mp−^) *S. salivarius* K12 strains. Approximately 1.5 × 10^6^ CFU pneumococci were added to D562 monolayers at an MOI of 10:1. Pneumococcal adherence was determined when incubated with pneumococci alone (Pnc, normalised to 100 %), or pre-incubated for 1 h with *S. salivarius* K12 or *S. salivarius* K12^mp^^-^ at high (~2 × 10^7^ CFU); medium (~2 × 10^6^ CFU, med); or low (~2 × 10^5^ CFU) doses. Data are mean + SD; *n* ≥ 6. * indicates *p* < 0.05, ** indicates *p* < 0.001 when compared to Pnc alone (Student’s t test)

**Fig. 4:**
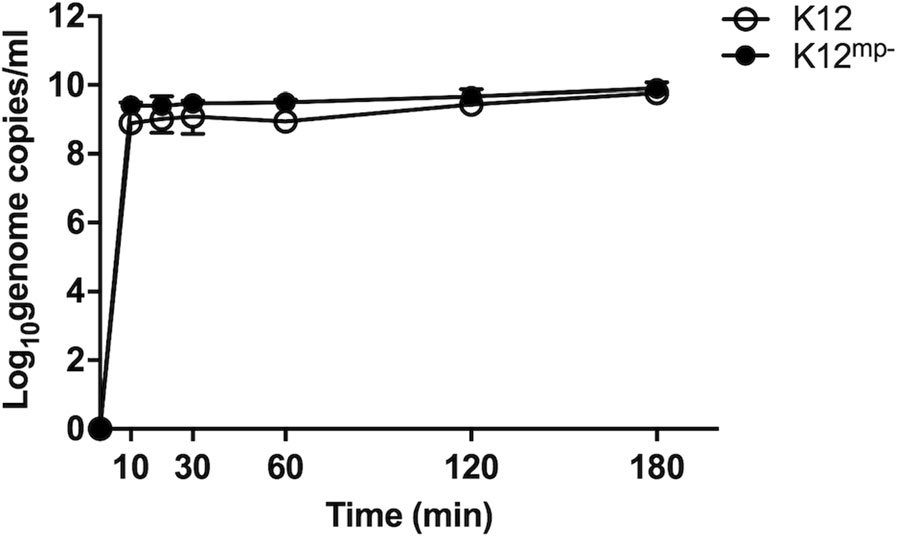
Time course of *S. salivarius* adherence to D562 cells. Cells were inoculated with either ~1.1 × 10^5^ CFU of *S. salivarius* K12 or ~1.9 × 10^5^ CFU of *S. salivarius* K12^mp-^ and the number of adherent bacteria measured over three hours. Median ± IQR for both *S. salivarius* isolates are depicted (*n* ≥ 2)

Since the *S. salivarius* megaplasmids were essential to prevent the growth of pneumococci, and K12 caused a greater reduction in pneumococcal adherence than M18 (Fig. 1), we next compared the ability of K12 and K12 ^mp−^ strains to inhibit pneumococcal adherence to D562 cells. Although the exact copy number of the megaplasmid is unknown, preliminary qPCR analysis confirmed that the ratio of K12 chromosomal DNA to megaplasmid DNA remained stable during bacterial growth (1.43, *n* = 1) and throughout assay conditions (1.17 and 1.20 after 1 and 2 h incubation with D562 monolayer, respectively, *n* = 1). All doses of K12 ^mp−^ significantly inhibited 6A adherence (high: *p* < 0.001, medium: *p* < 0.001, low: *p* = 0.016, Fig. 3a). When compared to K12, there was some evidence that the K12 ^mp−^ strain resulted in less inhibition than the K12 strain, but this difference was only statistically significant at the high dose (*p* = 0.044, Fig. 3a). For the 19F strain, only the high and medium doses of K12 ^mp−^ significantly inhibited pneumococcal adherence. We found no differences in the ability of K12 and K12 ^mp−^ to adhere to D562 cells (532 ± 86.49 % vs. 511 ± 6.92 %, *p* = 0.695) or in their adherence to D562 cells over time (Fig. 4, *p* > 0.05 for all time points).

### Cell contact is required for K12 to inhibit adherence by serotype 19F, but not serotype 6A

To determine whether contact of *S. salivarius* with D562 cells was required to inhibit pneumococcal adherence, we performed adherence assays in which K12 was administered using transwell inserts, preventing direct contact with epithelial cells but allowing the passage of secreted molecules. Using this transwell system, no inhibition of 19F adherence was observed at any dose, while 6A adherence was inhibited at the medium (*p* = 0.049) and low (*p* < 0.001) doses (Table 3). Preliminary experiments showed that culture supernatants did not inhibit pneumococcal growth in the deferred antagonism test.

### Protein synthesis may be required for K12 to inhibit 6A adherence

**Fig. 5:**
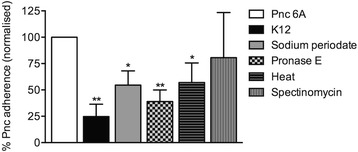
Adherence of pneumococcal serotype 6A to pharyngeal epithelial cells following pre-administration of treated *S. salivarius* K12. Approximately 1.5 × 10^6^ CFU pneumococci were added to D562 monolayers at an MOI of 10:1. Pneumococcal adherence was determined when incubated with pneumococci alone (Pnc, normalised to 100 %), or pre-incubated for 1 h with approximately 1.5 × 10^7^ CFU *S. salivarius* K12 (untreated), or treated with sodium periodate, Pronase E, heat-killed (heat), or spectinomycin. Data are mean + SD; *n* ≥ 3. * indicates *p* < 0.05, ** indicates *p* < 0.001 when compared to Pnc alone (Student’s t test)

The above experiments indicated the involvement of multiple mechanisms for *S. salivarius* inhibiting pneumococcal adherence. We further investigated the molecular mechanism(s) of *S. salivarius* K12 inhibition of 6A adherence by performing different treatments on the K12 inoculum prior to its use in adherence assays. Denaturing proteins and killing K12 cells (heat-treatment), interrupting protein synthesis (spectinomycin treatment), and removing outer surface carbohydrates (sodium periodate treatment) and proteins (pronase E treatment) all reduced inhibition of 6A adherence compared with untreated K12 (Fig. 5). However, only spectinomycin-treated K12 could no longer significantly inhibit 6A adherence (*p* = 0.402, Fig. 5).

## Discussion

In this study, we found that various bacteriocin-producing strains of *S. salivarius* can prevent pneumococcal growth on solid media and that the commercial probiotic strains, K12 and M18, inhibit pneumococcal 6A and 19F adherence to a pharyngeal epithelial cell line. Studies investigating the probiotic mechanisms of *S. salivarius* have largely focused on the role of bacteriocins and BLIS. The ~190 kb megaplasmid of *S. salivarius* K12 harbours genes encoding the bacteriocins salivaricin A2 and B, which prevent the growth of some bacterial pathogens, including *Streptococcus pyogenes*, in vitro [7, 14, 15]. Our results showed that the K12 megaplasmid is required to prevent pneumococcal growth in vitro, but is not essential to inhibit pneumococcal adherence. Therefore the primary mechanism of inhibition of pneumococcal adherence in this model does not appear to be mediated by megaplasmid-encoded bacteriocins; although we note that lack of inhibition of 19F adherence by a low dose of K12^mp-^ indicates a possible role for the megaplasmid at this concentration. Instead, our findings indicated that *S. salivarius* K12 can inhibit pneumococcal adherence by blocking pneumococcal binding sites on D562 cells. We observed a correlation between *S. salivarius* K12 adherence to D562 cells and inhibition of adherence by pneumococcal isolate PMP843 (19F). We also found that K12 contact with cells was essential for this inhibition. K12 binding to D562 cells did not require megaplasmid-encoded molecules, since a megaplasmid-negative strain of K12 displayed equal adherence. We observed differences in the inhibition of pneumococcal adherence depending on both the strain of *S. salivarius* used and the pneumococcal isolate tested. This was not unexpected, given that *S. salivarius* strains vary in their capacity to adhere to epithelial cells and inhibit other pathogens [7, 15, 16]. The pneumococcus is a diverse pathogen, with different strains and serotypes known to behave differently in vitro and in vivo [25, 26]. Overall, *S. salivarius* K12 displayed stronger inhibition of pneumococcal adherence than the M18 strain, possibly due to its increased capacity to adhere to D562 cells. Interestingly, K12 adherence to D562 cells did not correlate with 6A adherence, suggesting that mechanisms other than *S. salivarius*-mediated blocking of pneumococcal binding sites on epithelial cells may be involved in the inhibition of 6A adherence. This was further supported by the inhibition of 6A adherence by all doses of M18 despite its low adherence capability. Although a high dose of megaplasmid-negative K12 could still inhibit 6A adherence, the effect was significantly less than that observed with megaplasmid-positive K12, indicating a possible contribution to inhibition by the K12 megaplasmid in this assay. Transwell experiments demonstrated that for 19F, contact of *S. salivarius* K12 with D562 cells was required to inhibit pneumococcal adherence, consistent with a previous report showing that contact with epithelial cells was needed for *S. salivarius* K12 to inhibit adherence by *S. pyogenes* to HEp-2 cells [27]. However, low doses of K12 administered in the absence of cell contact could inhibit adherence of 6A. This could be due to bacteriocins or other secreted products, which may not necessarily be encoded on the megaplasmid. The differences observed using varying doses of probiotic suggest that quorum sensing may play a role in the expression of *S. salivarius* genes relevant to pneumococcal inhibition.

Further transwell experiments that test the K12 megaplasmid-negative strain may determine whether the molecules involved are chromosomally or plasmid encoded. Preliminary attempts to detect if bacteriocins were secreted through transwell membranes by deferred antagonism were unsuccessful, however this approach may have been insufficiently sensitive.

Overall, the data obtained in this study suggest that the primary mechanism by which *S. salivarius* K12 inhibits pneumococcal adherence to D562 cells is by blocking pneumococcal attachment to the epithelial cell surface. A number of pneumococcal surface molecules have been shown to play a role in adherence, such as the adhesion molecule PsaA, which binds to cell surface carbohydrates [28]. For *S. salivarius*, various cell surface molecules including fibrils and fimbriae have been implicated in adherence [29]. As heparin does not block *S. salivarius* adherence to D562 cells, glycosylaminoglycans do not seem to be a shared cell surface receptor. Since airway epithelial cells derived from carcinomas, such as D562 cells, can display altered expression of surface molecules compared to primary human epithelial cells [30], further investigation of common receptors used by these species should include testing of primary cells. Although inhibition of pneumococcal adherence could be due to competition for receptor-mediated binding, it could also result through a less specific mechanism, such as steric hindrance, or the ability of *S. salivarius* to mask pneumococcal binding sites. Other potential mechanisms by which *S. salivarius* may inhibit pneumococcal adherence, such as immune modulation of epithelial cells [31], were not examined in this study. Additionally, the in vitro model lacks the complexity of the nasopharynx, which can harbour a wide range of colonising bacterial species that interact with each other and with epithelial cells.

Whilst megaplasmid-encoded bacteriocins are required to inhibit the growth of pneumococci on solid media, our data suggest that it is the adherence of *S. salivarius* to epithelial cells that primarily blocks pneumococcal adherence in vitro. Therefore, strategies to use *S. salivarius* as a probiotic in the respiratory tract should optimise the ability of this species to colonise the target tissue. In vitro studies performed in our laboratory, and by others, have shown that the addition of probiotic bacteria to epithelial cells prior to pathogen administration is more effective than attempting to disrupt established pathogen colonisation [17, 27]. In clinical studies, high doses of M18 achieve greater colonisation rates and density in the oral cavity compared to lower doses [15]. Clinical studies of *S. salivarius* K12 as an oral probiotic have involved daily administration of high doses of the bacterium in an effort to achieve long-term colonisation of the oral cavity [32–34]. *S. salivarius* may be better suited as a probiotic for the oral cavity, where it predominates, than for the nasopharynx, the preferred niche of the pneumococcus. Strategies that facilitate *S. salivarius* adherence to the nasopharyngeal epithelium, such as the nasal spray recently employed by Santagati and colleagues [35], would likely be needed for this probiotic to inhibit pneumococcal colonisation in vivo.

## Conclusions

Bacteriocin-encoding megaplasmids of *S. salivarius* strains K12 and M18 were essential to prevent pneumococcal growth on solid media but were not required to inhibit pneumococcal adherence to pharyngeal epithelial cells. Our results suggest that *S. salivarius* K12 employs several mechanisms, including blocking pneumococcal binding sites, to reduce pneumococcal adherence to pharyngeal epithelial cells. Further research is needed to identify the specific molecules involved. These findings contribute to our understanding of how probiotics may inhibit pneumococcal adherence and could assist with the development of novel strategies to prevent pneumococcal colonisation in the future.

## Additional files

Additional file 1: Supporting Information S1. Raw data used to generate figures and tables. (XLSX 52 kb) Additional file 2: Table S1. Specificity of *dex* and *mp* gene targets to detect *Streptococcus salivarius*. DNA was extracted from pure cultures grown on HBA agar and 0.1 ng DNA of each isolate listed was used as a template for the *dex* and *mp* qPCR reactions, as described in the methods section. Mean Ct from duplicate wells is shown. (DOCX 15 kb)

## Abbreviations

ATCC: American type culture collection
BLIS: Bacteriocin-Like Inhibitory Substance
CFU: Colony forming unit
DNA: deoxyribonucleic acid
EDTA: Ethylenediaminetetraacetic acid
FBS: Foetal bovine serum
HBA: Horse blood agar
HBSS: Hanks buffered salt solution
MOI: Multiplicity of infection
PCR: Polymerase chain reaction
THB: Todd Hewitt broth
THY: Todd Hewitt broth supplemented with yeast extract
