# Supplementary material for: Investigation of Streptococcus salivarius-mediated inhibition of pneumococcal adherence to pharyngeal epithelial cells
Source: BMC Microbiol. 2016 Sep 29;16:225. doi: 10.1186/s12866-016-0843-z (PMC5041332; doi:10.1186/s12866-016-0843-z)
Supplement: Additional file 2: Table S1. — Specificity of dex and mp gene targets to detect Streptococcus salivarius. DNA was extracted from pure cultures grown on HBA agar and 0.1 ng DNA of each isolate listed was used as a template for the dex and mp qPCR reactions, as described in the methods section. Mean Ct from duplicate wells is shown. (DOCX 15 kb) [file 12866_2016_843_MOESM2_ESM.docx]

**Table S1. Specificity of *dex* and *mp* gene targets to detect *Streptococcus salivarius*.**

| **Isolate** | | **Mean Ct** | |
| --- | --- | --- | --- |
| **Species** | **Strain** | ***dex*** | ***mp*** |
| *Streptococcus salivarius* | K12 | 21.61 | 22.35 |
| *S. salivarius* | K12^mp-^ | 22.43 | No Ct |
| *S. salivarius* | M18 | 22.44 | 23.20 |
| *S. salivarius* | M18 ^mp-^ | 20.26 | No Ct |
| *Streptococcus pneumoniae* | PMP1043 | No Ct | No Ct |
| *S. pneumoniae* | PMP843 | No Ct | No Ct |
| *Streptococcus mitis* | PMP933 | No Ct | No Ct |
| *Streptococcus mutans* | PMP935 | No Ct | No Ct |
| *Streptococcus sanguis* | PMP936 | No Ct | No Ct |
| *Streptococcus anginosus* | PMP1049 | No Ct | No Ct |
| *Streptococcus dysgalactiae* | PMP1051 | No Ct | No Ct |
| *Streptococcus sobrinus* | PMP1053 | No Ct | No Ct |
| *Streptococcus cricetus* | ATCC 19642 | No Ct | No Ct |
| *Streptococcus oralis* | PMP1056 | No Ct | No Ct |
| *Streptococcus gordonii* | PMP1057 | No Ct | No Ct |
| *Streptococcus agalactiae* | PMP994 | No Ct | No Ct |
| *Streptococcus pyogenes* | PMP1000 | No Ct | No Ct |
| *Streptococcus vestibularis* | PMP1059 | No Ct | No Ct |
| *Streptococcus infantis* | PMP1301 | No Ct | No Ct |
| *Streptococcus australis* | PMP1303 | No Ct | No Ct |
| *Streptococcus* *cristatus* | PMP1306 | No Ct | No Ct |
